# Are cognitive subtleties too subtle to see? A diffusion tensor imaging validation of the subtle cognitive impairment test and other psychometric assessments in a normative sample

**DOI:** 10.1007/s11682-026-01139-5

**Published:** 2026-04-07

**Authors:** Iain D. Croall, Paul A. Armitage, Marios Hadjivassiliou, Nigel Hoggard

**Affiliations:** 1 School of Medicine and Population Health, Royal Hallamshire Hospital, University of Sheffield, University MRI, C Floor, Glossop Road, Sheffield, S10 2JF UK; 2 Department of Neurology, Sheffield Teaching Hospitals NHS Foundation Trust, Sheffield, UK

**Keywords:** Diffusion tensor imaging, Dementia, Mild cognitive impairment, Subjective cognitive decline, Subtle cognitive impairment test

## Abstract

Dementia care and research is limited by difficulties identifying patients in its prodrome. Existing cognitive screening forms have poor sensitivity with respect to subtle changes. The Subtle Cognitive Impairment Test has been proposed as a superior assessment, but remains largely unproved. We investigate this and other cognitive tests by establishing their sensitivity to brain diffusion tensor imaging metrics in a sample of healthy, older adults. Healthy participants (*N* = 84) aged 50–70 were recruited. All subjects underwent a holistic cognitive test battery. Diffusion tensor imaging data were processed to create maps including mean diffusivity, fractional anisotropy, radial and axial diffusivity. Cross-sectional correlations compared mean diffusivity and fractional anisotropy against all cognitive test outcomes using tract-based spatial statistics, with any significant results being re-run with age correction. Models which survived this were further inspected with regards to axial and radial diffusivity as appropriate. Only the subtle cognitive impairment test (“tail percentage errors”) and trail making (“A” condition) provided areas where voxels reached p < 0.05 in univariate models, while only the former survived age correction. Post-hoc analyses using the subtle cognitive impairment test variable found a small cluster of significant voxels with respect to axial diffusivity. It appears unaffected by participant level of education, mood or quality of life. The subtle cognitive impairment test appears to be a superior cognitive marker of subtle shifts in neurophysiology and may therefore be a desirable tool in the investigation or triage of early neurodegeneration.

**Supplementary Information:**

The online version contains supplementary material available at 10.1007/s11682-026-01139-5.

## Introduction

Dementia care and research is restricted by the difficulty of identifying the condition in its prodrome. Currently Alzheimer’s disease may not be diagnosed for over 15 years after relevant biomarkers are detectable (Dubois et al. 2016). Such late identification means that patients are unable to start available treatments until the disease has reached a relatively advanced stage; this represents a particularly catastrophic missed opportunity in subtypes such as vascular dementia where progression is modifiable with a mixture of clinical and lifestyle interventions (Livingston et al. 2020). It is also a limiting factor in research of the condition as studies are unable to recruit and investigate its early course, where the most important understanding may be gained and the most benefit may be seen in randomised trials.

Diffusion Tensor Imaging (DTI) is an MRI technique which is adept at detecting early, dementia-related neurophysiological changes (Chen et al. 2023). It quantifies the microstructural health of brain white matter and has been shown to discern between participants with Subjective Cognitive Decline (SCD) and healthy controls (Brueggen et al. 2019, Li et al. 2016). DTI predicts development of dementia across a range of neurological health (Egle et al. 2022), and is accordingly used as an outcome in dementia clinical trials (Markus et al. 2021, Stephen et al. 2020).

Clinically, dementia diagnosis relies heavily on measuring cognitive ability where validated, short-form cognitive tests such as the Mini-Mental State Exam (MMSE (Folstein et al. 1975)) and Montreal Cognitive Assessment (MoCA (Nasreddine et al. 2005)) are common. However, these tests lack effectiveness at detecting early cognitive decline. Mild Cognitive Impairment (MCI) often indicates a developing dementia (Mariani et al. 2007), but a recent meta-analysis found that the MoCA has only modest sensitivity (73.5–83.8%, depending on cut-off) and specificity (70.8–91.3%) for identifying this (Islam et al. 2023). A meta-analysis of the MMSE found poorer accuracy than this with pooled sensitivity/specificities of 63.4%/65.4% (Mitchell 2009).

MCI has no consensus criteria, but as suggested models for it generally require some “objective” features for diagnosis (e.g. set deviations from normative memory test performance) (Bradfield 2023) this excludes people who self-report cognitive problems but who still perform relatively well on psychometric tests. The aforementioned SCD generally refers to this more subjective phenomenon (Jessen et al. 2020), and arguably represents the earliest phase of a dementia where an impact on cognitive ability would become theoretically measurable. While SCD can be driven by anxiety or hypersensitivity, studies support that in many individuals the presentation is a manifestation of early-stage dementia (Mitchell et al. 2014). Existing cognitive tests do have some utility in studying subtle deficit, but reports demonstrating this typically combine multiple assessments into a composite score (Shen et al. 2021). This makes their administration and interpretation more challenging, and impractical for a typical clinical environment.

The Subtle Cognitive Impairment Test (SCIT) (Yelland et al. 2006) has been proposed as a single assessment capable of measuring slight cognitive deterioration. It is a computer-based task that takes approximately 10 min to finish where the participant uses the keyboard to indicate if the left or right side of a shape (which either resembles a “U” or “H”) has a shorter stalk. The shapes are presented at various lengths of time but are only visible for brief or extremely brief intervals. Both the accuracy and timing of participant responses are measured. Studies using the SCIT have so far shown it discriminates experimentally between marginal variations in otherwise-normal MMSE scores (Friedman et al. 2011) and it is well correlated to various other cognitive tests (Bruce et al. 2014). These offer some support to the usefulness of SCIT but further validation is needed. Notably, research which links SCIT performance with markers of neurophysiology, such as neuroimaging, are lacking entirely.

In this study we investigate healthy, older volunteers. We compare the experimental sensitivity of performance on various common psychometric tests, including the SCIT, against DTI brain scans. By doing this we aim to provide data on the relative usefulness of different cognitive assessment options with respect to neuroimaging data which is known to be highly relevant to early dementia changes. Conducting this in controls focuses the investigation within the spectrum of performance and health which is considered ostensibly normal, presenting a challenging analysis where only subtle patterns of neurological and cognitive change would be available for study. Our hypothesis is that SCIT data will yield greater experimental sensitivity in this objective than other tests.

## Methods

### Study design and participants

This is a cross-sectional experiment of healthy volunteers, which uses data collected as part of a project which investigated if participants with a gluten-related antibody showed any signs of neurocognitive deficit compared to participants without this antibody. Those comparisons were ultimately negative (Croall et al. 2024) and so for this report all subjects are considered as controls.

Volunteers from the general public were recruited. The recruitment process and full participant pathway/criteria are reported elsewhere (Croall et al. 2024). Briefly, all subjects were aged between 50 and 70 and were free from any notable medical diagnoses, with particular attention being placed on anything that may impact the brain. Current smokers were excluded and no subject had accumulated more than 20 pack years of previous smoking. The current analyses focus only on those volunteers who completed cognitive testing and brain MRI scanning, which were administered together during the same data collection visit.

Ethics for the study were approved by a University of Sheffield ethics committee (UREC #030250).

### Cognitive Testing and Questionnaires

Cognitive tests were delivered in a single session and always by the same investigator. These were a holistic combination of psychometric tests used in research and clinical settings. The delivery order of the tests (Strauss et al. 2006), their purpose if not already defined, and any key outcomes used in analyses were as follows:

1. The National Adult Reading Test (NART, premorbid IQ).
2. Rey-Osterrieth Complex Figure Test (visuospatial memory).

- Copy trial total errors.
- Immediate recall total correct.

3. Trail Making Test (TMT, processing/motor speed and task switching).

- A condition time.
- B-A condition time.

4. SCIT (Yelland et al. 2006): each outcome is separately analysed for “head” (fastest/most challenging) and “tail” (slowest/least challenging) stimulus presentations.

- Average response (ms).
- Percentage errors.
- N timeouts.

5. Rey-Osterrieth Complex Figure Test.

- Delayed recall total correct.

6. Digit Span (short term/working memory).

- Forwards total score.
- Backwards total score.

7. Letter Fluency (FAS cues, phonemic word generation).

- Total correct.

8. Category fluency (animals, semantic word generation).

- Total correct.

9. MoCA (Brueggen et al. 2019).

- Total score.

Following cognitive testing participants completed a set of questionnaires which included the Hospital Anxiety and Depression Scale (HADS) (Zigmond and Snaith 1983) and the SF-36 (Ware & Sherbourne 1992) to measure quality of life.

### MRI scanning

MRI scans tool place immediately after cognitive testing. Subjects were scanned on a 3 T GE SIGNA PET/MR (GE Healthcare, Milwaukee, WI) with a 32-channel coil (Nova Medical, Wilmington, MA). Including all sequences the acquisition lasted 30 min. Relevant to the current paper the protocol included a DTI scan with 50 directions (b = 1000), 5 b0 images, a 2 mm isotropic resolution, 75 slices, 25.6 cm FOV, and a TR/TE = 7.1040/0.0770s. An additional b0 image was collected with a reversed phase direction for geometric distortion correction.

### Image processing

DTI scans were processed with the objective of performing Tract-Based Spatial Statistics (TBSS) (Smith et al. 2006) analyses. After conversion to NifTI data format using “dcm2niix” all tools used were part of “FSL” (FMRIB’s Software Library (Smith et al. 2004)) pipelines. Geometric distortion and motion correction was performed on the raw data using TOPUP and EDDY, before maps of Fractional Anisotropy (FA), Mean Diffusivity (MD), Axial Diffusivity (AD) and Radial Diffusivity (RD) were calculated using DTIFIT. Raw and processed images were visually inspected for quality and for all subjects deemed suitable to proceed to main analyses. TBSS was then used to register FA maps into a standard space and project central tract values onto the FA “skeleton”, which was thresholded using the default value of 0.2. These registrations were then separately applied to the MD, AD and RD data.

### Statistical analyses

A series of exploratory analyses were first conducted wherein all key cognitive test outcomes, and age, were separately correlated against the TBSS-processed FA and MD maps via using FSL’s RANDOMISE with 500 permutations and Threshold-Free Cluster Enhancement (TFCE) correlation applied. Given the small number of permutations these were considered as “pilot” analyses, and where any *p* values < 0.1 were achieved that analysis was then repeated with 10,000 permutations for confirmation. Significant results from this (i.e. where *p* < 0.05) were further re-run with age correction applied. In order to compare the relative sensitivity of different cognitive tests with respect to the DTI data, all significant findings from univariate and multivariate (age-corrected) analyses were summarised by both the lowest achieved *p* value and the number of voxels which reached statistical significance.

Any such models achieving significance with age-correction underwent further TBSS studies investigating AD and RD. Similarly, any cognitive tests that held significant, age-corrected associations with any DTI measure were explored via correlations with common confounding factors of age, level of education, NART errors, body mass index, HADS depression/anxiety and SF-36 outcomes.

Any subjects with any missing data were excluded from all analyses so that results would be fully-comparable within the same cohort.

## Results

84 subjects were included in analyses. Their demographic and other data are summarised in Table 1.

**Table 1:** Summary of demographic data of the 84 subjects included in analyses

Variable	Summary
Age (mean ± SD)	60.4 ± 6.0
Sex (% female)	78.6%
BMI (mean ± SD)	25.6 ± 3.8
Highest level of education (approximate USA equivalent/further description)	**GCSE** (high school diploma): 13.1% **A Level** (advanced placement course): 9.5% **Diploma** (intermediary vocational qualification): 31.0% **Undergraduate degree**: 21.4% **Masters degree**: 19.0% **PhD**: 6.0%
Smoking history	**Never smoked**: 63.1% **Previous smoker**: 36.9%
Hypertension (% yes)	11.9%

*BMI* Body Mass Index

Pilot TBSS investigations found four cognitive outcomes to potentially correlate with DTI FA and MD (i.e. resulted in at least one cluster where the minimum *p* value was < 0.1). Trail Making A (TMTA) and SCIT percentage errors (head conditions) held potentially significant relationships with both FA and MD, while forwards digit span and SCIT percentage errors (tail conditions) held potentially significant relationships with FA only. Strong correlations between age and FA/MD were also indicated. In all cases the directions of association indicated higher MD/lower FA to be associated with poorer cognition/greater age, except for digit span where worse performance was provisionally linked to higher FA.

Full experimental analyses confirmed that FA held statistically significant associations with SCIT percentage errors (tail conditions, Supplementary Fig. 1) and TMTA (Supplementary Fig. 2), while TMTA was also shown to significantly correlate with MD (Supplementary Fig. 3). Age was also confirmed to significantly relate to FA/MD. Further details of these models are shown in Table 2.

**Table 2:** Summary statistics for the three cognitive models which provided significant clusters in TBSS analyses, in both univariate models and with age correction applied

Model	Lowest *p* value	N voxels with *p* < 0.05	% voxels with *p* < 0.05
FA vs. SCIT percentage errors (tail conditions)	*0.008*	16,472	10.5%
With age correction	*0.026*	3897	2.49%
FA vs. TMTA	*0.003*	36,915	23.6%
With age correction	*0.053*	-	-
MD vs. TMTA	*0.027*	9,834	6.3%
With age correction	*0.205*	-	-

*FA* Fractional Anisotropy, *MD* Mean Diffusivity, *SCIT* Subtle Cognitive Impairment Test, *TBSS* Tract-Based Spatial Statistics, *TMTA* Trail Making Test (“A” condition)

**Fig. 1:**
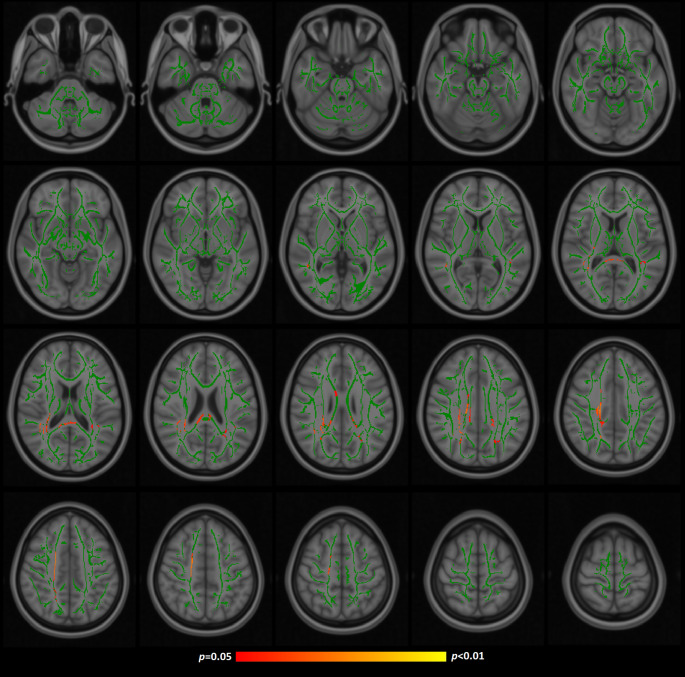
TBSS areas of negative correlation between FA and SCIT percentage errors (tail conditions) following age-correction

**Fig. 2:**
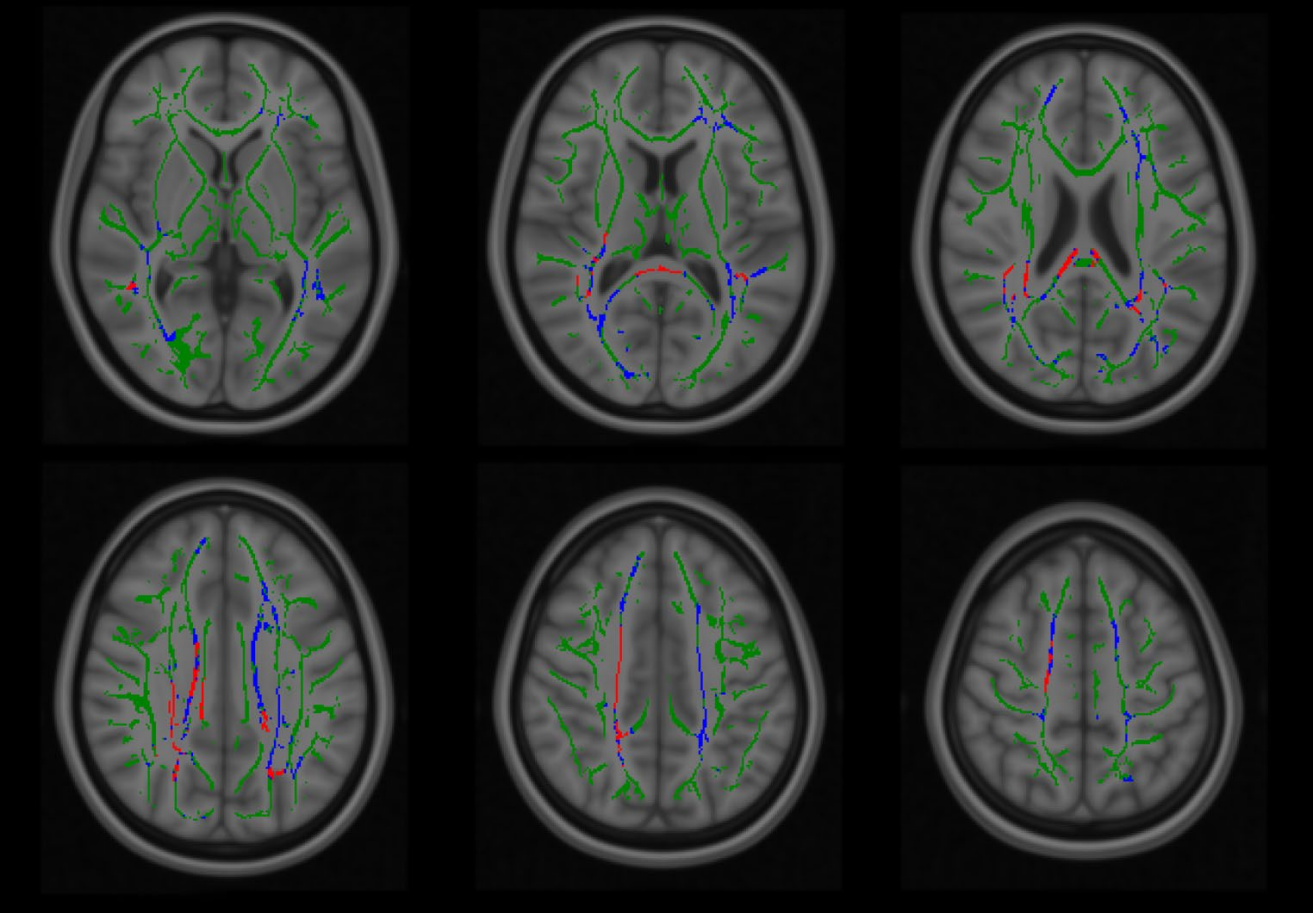
A comparison between areas which achieved significant correlation between FA and SCIT percentage errors (tail conditions), without age correction (blue regions) and the reduction in regional significance with age correction applied (red regions). Only those areas which showed any age-corrected significant results are included in these panels

Without age correction the strongest appearing cognitive model was between FA and TMTA, which provided the cluster with the overall lowest *p* value (0.003) and also the greatest overall number of significant voxels. However, when repeated with age correction only the association between FA and SCIT percentage errors (tail conditions) survived, with 2.49% of voxels reaching significance and an overall lowest *p* value of 0.026. The JHU ICBM DTI 81 (white matter labels) atlas (Mori et al. 2008) indicated that these results included the posterior thalamic radiation, splenium and body of the corpus callosum, superior longitudinal fasciculus, corona radiata, superior corona radiata and cingulum. See Figs. 1 and 2. TMTA scores “approached” significance in these models with respect to FA (lowest *p* = 0.053) and were not significant when analysed with respect to MD (lowest *p* = 0.205).

**Fig. 3:**
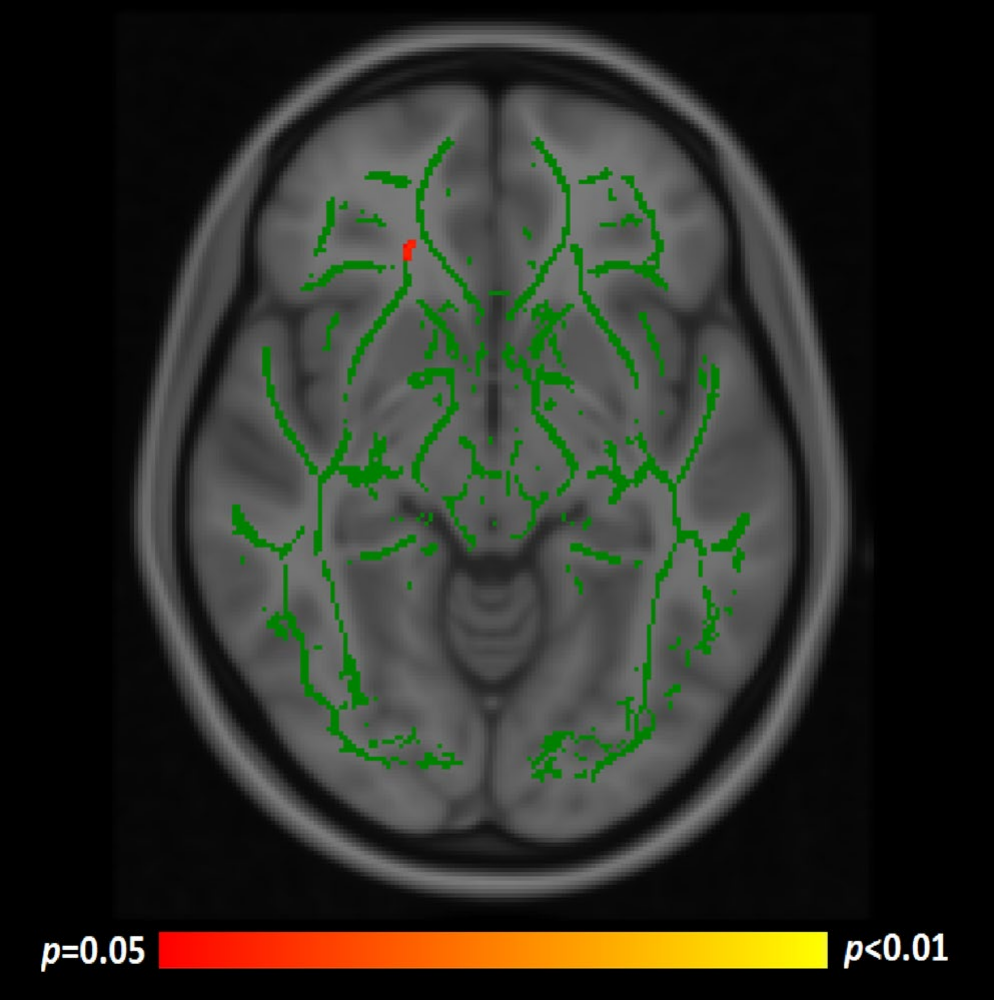
A single cluster of significant negative correlation between AD and SCIT percentage errors (tail conditions) following age-correction

Given the significant age-corrected finding between FA and the SCIT outcome, post-hoc TBSS analyses were performed investigating for any associations with AD/RD. AD provided one negatively-correlating cluster (age-corrected) vs. SCIT percentage errors (tail conditions), showing greater AD to predict fewer test errors. This is visualised in Fig. 3. The JHU atlas indicated this to be in the anterior corona radiata. There were no significant correlations between RD and SCIT performance.

Finally, for the benefit of future experimentation exploratory analyses were performed to examine if SCIT percentage errors (tail conditions) held any associations with variables which may be considered potentially confounding or otherwise of interest. As the SCIT outcome was not normally distributed all tests are non-parametric. Spearman correlations confirmed a significant positive correlation with age (*r* = 0.250, *p* = 0.022) i.e. suggesting worsening performance with greater age. However, it did not correlate with NART performance (as a marker of IQ/education), body mass index, HADS anxiety/depression (raw scores) or any of the 8 conventional SF-36 outcomes. A Kruskal Wallis analysis directly comparing levels of education was not significant.

## Discussion

In this study of healthy, older volunteers, we investigated the experimental sensitivity of various cognitive assessments with respect to brain DTI patterns. Of these, only the TMTA and the percentage errors outcome of the SCIT (for “tail” conditions) significantly correlated with DTI outcomes, while only the SCIT variable survived age correction. This provides support that the SCIT test may be a superior tool for detecting small neurophysiological variations.

There is an increasing need for cognitive tests which can detect subtle neurophysiological deterioration. Such tools, if they are also of short duration and easy administration, would allow for practical measurement of early neurodegeneration and injury. This would be highly valuable for dementia care and research as detecting the condition’s prodrome presents a substantial challenge. The prevalence of conditions such as MCI and SCD, where true cases of early cognitive impairment may be masked by subjective patient anxieties, further adds to the need for accurate measurement of slight cognitive deteriorations.

Research in this area is dominated by studies of MCI and SCD, but these investigations face challenges in uncertain participant definitions and the difficulty of longitudinal follow-up that is needed to identify true cases. An approach that has seldom been employed is to study healthy, older volunteers. We would expect from such a group a tight spectrum of neurophysiological health driven by differences in lifestyle risk factors (Livingston et al. 2020) etc., albeit all within “normative” bands. This therefore acts as a challenging scenario for any cognitive assessment to prove experimental sensitivity at detecting subtle, dementia-related neurological changes.

In our study we used DTI as a marker of underlying neurological health. DTI quantifies integrity of the brain’s white matter and is popular within research given it is adept at measuring small changes relevant to early dementia (Chen et al. 2023). It predicts dementia conversion (Egle et al. 2022) and is accordingly used as an outcome in dementia clinical trials (Markus et al. 2021, Stephen et al. 2020) and has also been shown capable of measuring differences between participants with SCD and matched controls (Brueggen et al. 2019, Li et al. 2016). This makes it an ideal benchmark indication of dementia-related brain health.

Seeking correlations between conventional DTI outcomes and cognitive performance, the TMTA and the SCIT percentage errors (tails) produced significant associations with FA (and in the case of the TMTA also MD). However, only the SCIT variable survived age correction. Age correction here is an essential step in order to minimise age-related variability and thus maximise the core effect of underlying neurophysiological health. It should be noted that the TMTA model did nearly achieve significance with age correction, though this nonetheless supports SCIT tail percentage errors as being the overall superior cognitive variable with respect to health of white matter microstructure. All relationships were in conventional directions implying decreasing FA/increasing MD to indicate worsening tract integrity. Post-hoc TBSS analysis investigating AD and RD found a single, small significant cluster with respect to AD. While this may suggest that SCIT tail percentage errors more specifically describes changes along the axon rather than perpendicular to it (which may indicate e.g. axonal injury rather than demyelination (Winklewski et al. 2018)), given the relative lack of post hoc findings this more likely suggests that AD and RD changes contribute relatively equally to the parent FA findings.

The SCIT was patented in 2006^18^ and has been used in a small number of research studies (Friedman et al. 2011, Bruce et al. 2014, Jianqin et al. 2016, Yelland 2017), though it has never been employed in any study alongside neuroimaging techniques. Sharing similarities to the Inspection Time task (Vickers et al. 1972), it is made a challenging assessment by short to very-short stimuli exposure times and principally relies on visual perception. Six outcomes are made available and test documentation suggests that each describe different aspects of brain health (Neurotest 2024). Our study indicates that of these results, the error rate is most effective at being a marker of white matter health, with the error rate of the tail (i.e. easier) stimuli being the best overall. Such significant (age corrected) tract regions were relatively widespread and went beyond immediate visual areas to include major areas such as the corpus callosum, suggesting tail percentage errors offer a holistic measurement of white matter integrity. While a significant relationship with age was found, post hoc analyses indicated that SCIT tail percentage errors did not correlate with level of education, mood or quality of life, implying its association with FA to be robust against common confounding factors.

Overall, this study is therefore supportive of the SCIT as being a potentially important tool for assessing cognitive differences with subtle underlying neurophysiological deficit. Coupled with its ease of administration and short completion time it may be a desirable cognitive tool for dementia and related research areas. Administration time could be further shortened if only the tail conditions are used, though this would require further research to be confident that they consistently provide superior data to the head conditions.

This study also highlights the overall difficulty in finding cognitive assessments which are capable of providing sensitive results with respect to markers of brain health. The cognitive test battery included the MoCA, a clinically-validated dementia tool, and a number of other tests popular within neuropsychological research for their proven utility for investigating deficits. Indeed, there are numerous examples of these assessments relating to DTI across various clinical contexts (Qiao et al. 2021, Wang et al. 2024, Koo et al. 2020, Duru et al. 2024), but in the current study failed to achieve significant correlations in a healthy control sample where underlying brain health will have less variability.

Our study suffers from limitations. Age is arguably the most influential confounder in this investigation, and while it was controlled for as an additional variable in final analyses this will not be as reliable as handling it in the main study design (e.g. by only investigating healthy volunteers of the same age). Our findings would also benefit from longitudinal follow-up of the participants to confirm which go on to develop dementia/MCI. While knowing this would add some clinical validity, it should not however diminish the current interpretation of the SCIT assessment having been the most effective as a marker of white matter tract health (and the only which reached statistical significance) in a challenging experimental scenario.

This study has provided unique data comparing cognitive performance with brain DTI scans in a normative sample. We find that a majority of common neuropsychological tests fail to achieve statistical significance, but that the TMT (“A” condition) and SCIT (tail percentage errors) show some experimental utility, with the SCIT being the only one to also survive age correction in final analyses. Our data therefore supports that the SCIT may indeed be a preferential tool for the study or clinical triaging of subtle cognitive deficit.

## Supplementary Information

Below is the link to the electronic supplementary material.

Supplementary Material 1 (PNG 906 KB)

Supplementary Material 2 (PNG 909 KB)

Supplementary Material 3 (PNG 903 KB)

## Data Availability

The datasets generated during and/or analysed during the current study are not publicly available due to the study ethics not permitting access by third parties.
