# Supplementary figures and images for: Are cognitive subtleties too subtle to see? A diffusion tensor imaging validation of the subtle cognitive impairment test and other psychometric assessments in a normative sample
Source: Brain Imaging Behav. 2026 Apr 7;20(2):70. doi: 10.1007/s11682-026-01139-5 (PMC13056773; doi:10.1007/s11682-026-01139-5)

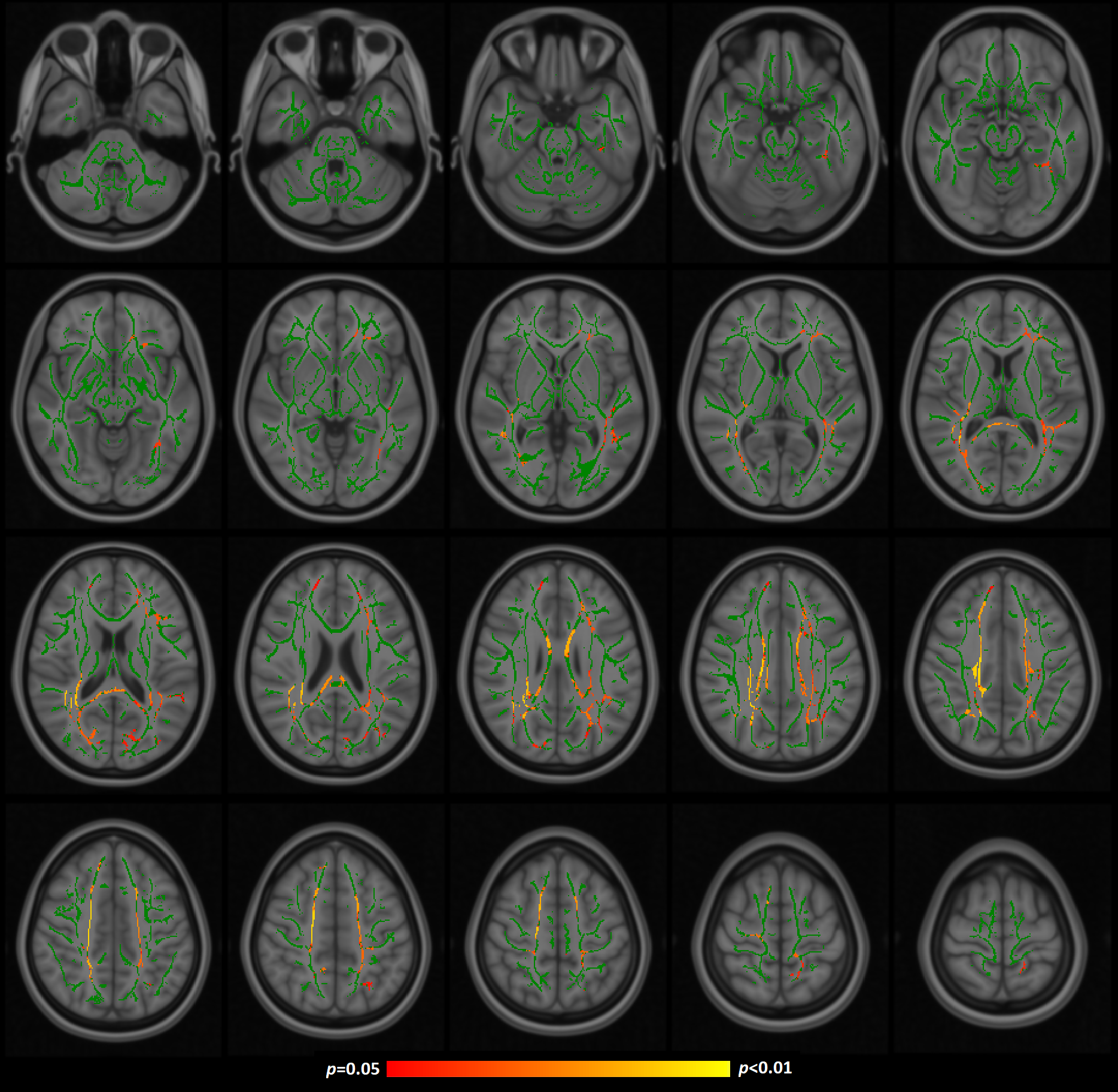

Supplement: Supplementary file 1 — Supplementary Material 1 (PNG 906 KB) [file 11682_2026_1139_MOESM1_ESM.png]

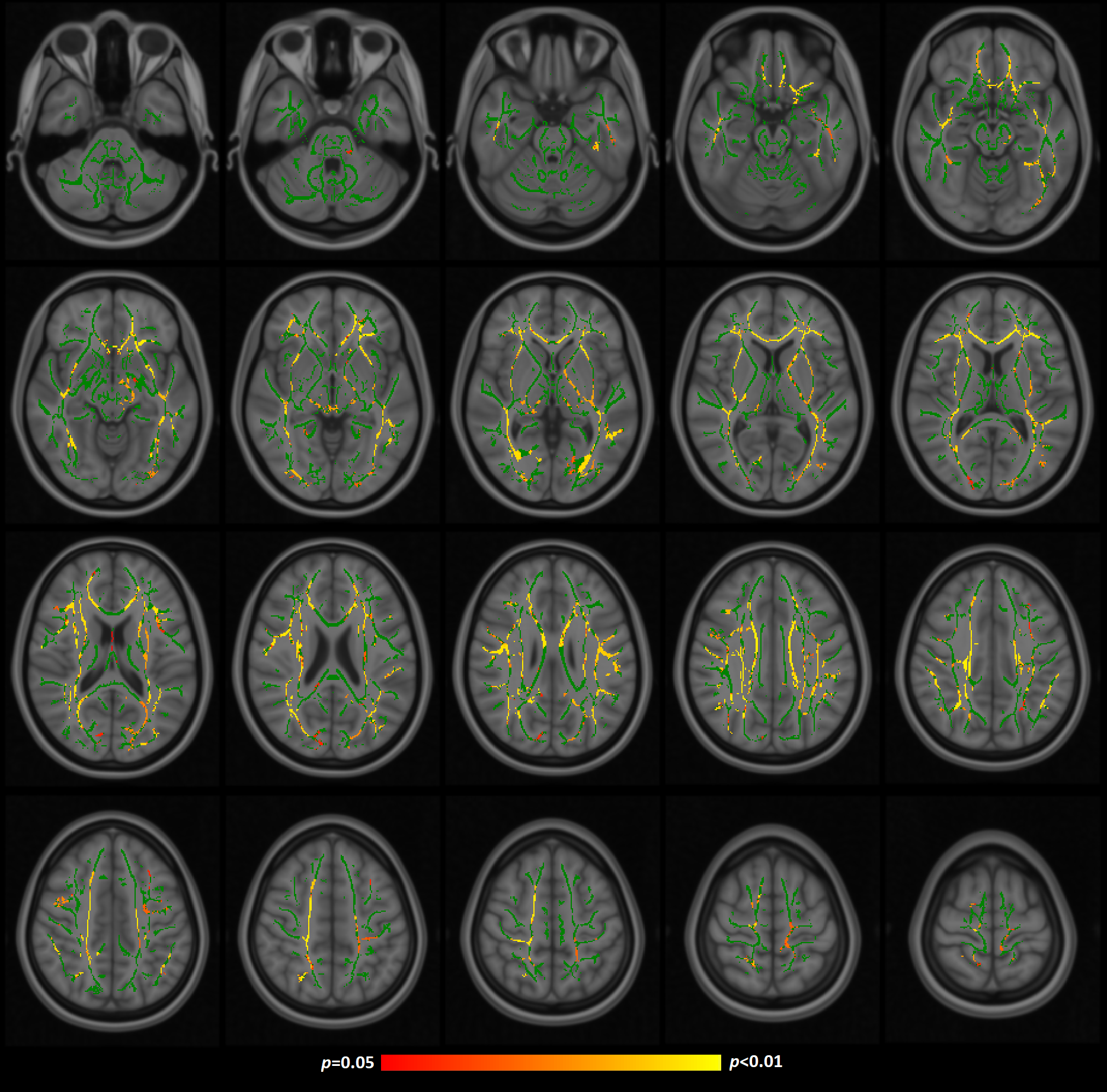

Supplement: Supplementary file 2 — Supplementary Material 2 (PNG 909 KB) [file 11682_2026_1139_MOESM2_ESM.png]

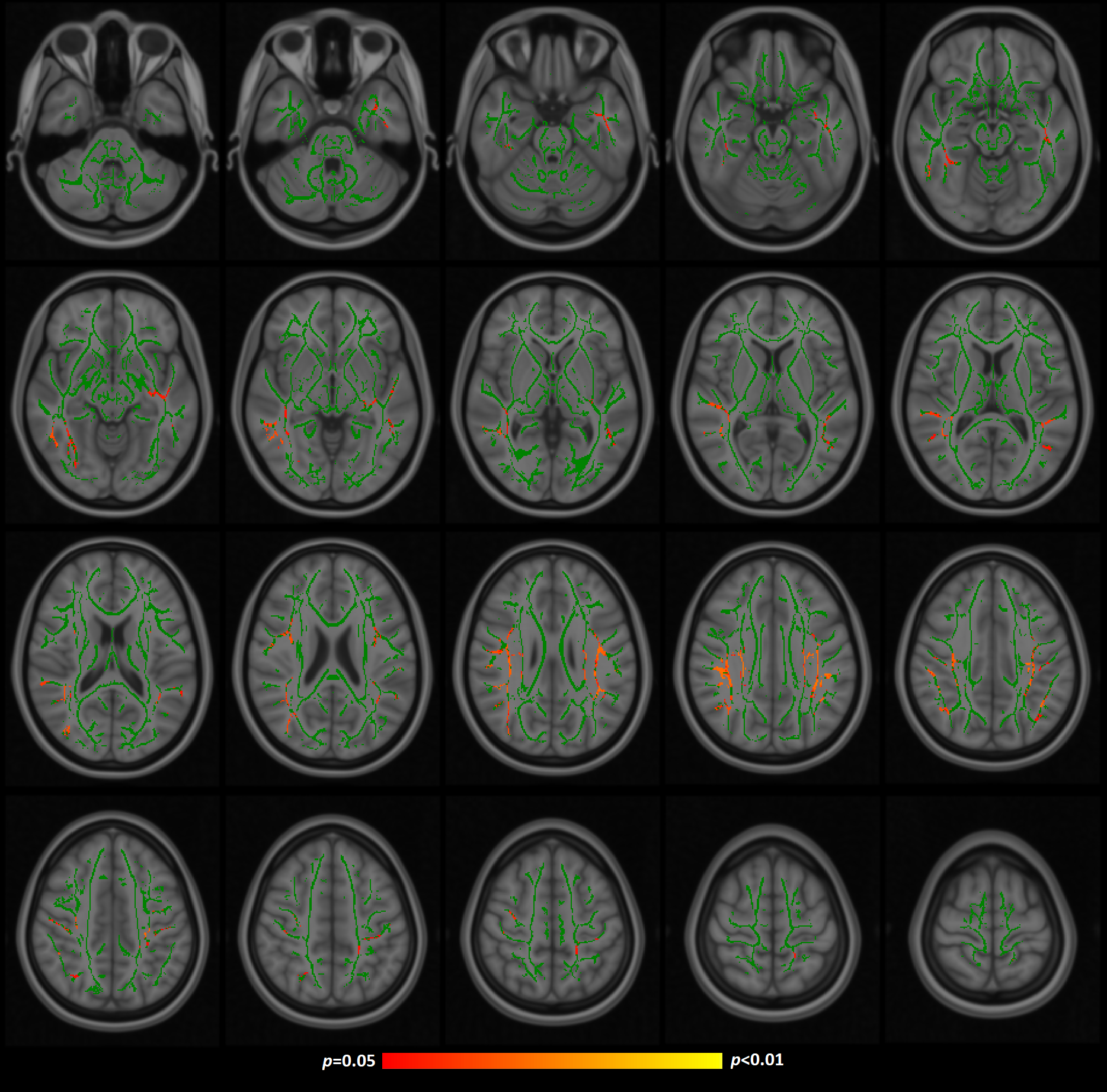

Supplement: Supplementary file 3 — Supplementary Material 3 (PNG 903 KB) [file 11682_2026_1139_MOESM3_ESM.png]
